# 2-Bromo-1,3-diisopropyl-4,5-dimethyl-1*H*-imidazol-3-ium dicyanidoargentate

**DOI:** 10.1107/S1600536811051828

**Published:** 2011-12-07

**Authors:** Eyad Mallah, Kamal Sweidan, Qutaiba Abu-Salem, Wael Abu Dayyih, Manfred Steimann

**Affiliations:** a Faculty of Pharmacy and Medical Sciences, Petra University, PO Box 961343, Amman 11196, Jordan; b Department of Chemistry, Faculty of Science, The University of Jordan, Amman, Jordan; c Department of Chemistry, Faculty of Science, University of Al al-Bayt, Al-Mafraq, Jordan; d Institut für Anorganische Chemie der Universität Tübingen, Auf der Morgenstelle 18, D-72076 Tübingen, Germany

## Abstract

The title structure, (C_11_H_20_BrN_2_)[Ag(CN)_2_)], is built up from an approximately *C*_2*v*_-symmetric imidazolium cation and a nearly linear dicyanidoargentate anion [N—Ag—N = 176.6 (9)° and Ag—C—N = 178.8 (9) and 177.2 (11)°]. These two constituents are linked by a remarkably short inter­action between the Br atom of the imidazolium cation [C—Br = 1.85 (3) Å] and one N atom of the cyanidoargentate anion [Br⋯N = 2.96 (2) Å], which is much less than the sum of the van der Waals radii (3.40 Å). The crystal studied was twinned by merohedry.

## Related literature

For similar structures, see: Mallah *et al.* (2009 ▶, 2011 ▶); Kuhn *et al.* (2009 ▶); Potocenak & Chomic (2006 ▶); Mascal *et al.* (1996 ▶). For the synthesis of the starting material, see: Kuhn *et al.* (2004 ▶).
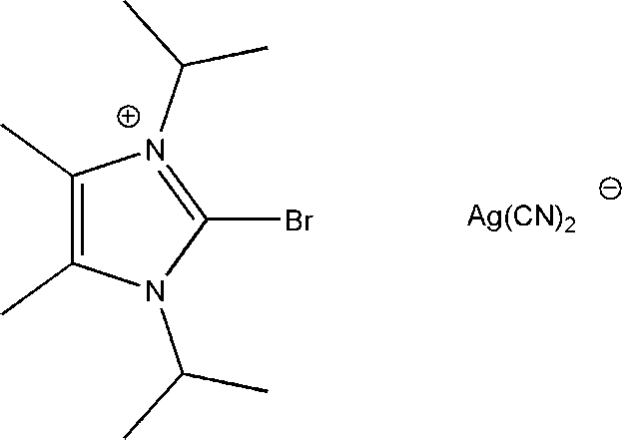


## Experimental

### 

#### Crystal data

(C_11_H_20_BrN_2_)[Ag(CN)_2_)]
*M* *_r_* = 420.11
Orthorhombic, 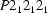

*a* = 6.6986 (15) Å
*b* = 10.6222 (14) Å
*c* = 23.989 (4) Å
*V* = 1706.9 (5) Å^3^
*Z* = 4
Mo *K*α radiation
μ = 3.52 mm^−1^
*T* = 291 K
0.40 × 0.20 × 0.20 mm

#### Data collection

Enraf–Nonius CAD-4 diffractometer
Absorption correction: refined from Δ*F* (*DIFABS*; Walker & Stuart, 1983 ▶)  *T*_min_ = 0.41, *T*_max_ = 1.00
4327 measured reflections
3475 independent reflections
1781 reflections with *I* > 2σ(*I*)
*R*_int_ = 0.052
3 standard reflections every 300 reflections  intensity decay: 1.5%

#### Refinement

*R*[*F* ^2^ > 2σ(*F* ^2^)] = 0.052
*wR*(*F* ^2^) = 0.082
*S* = 0.98
3475 reflections
180 parameters
H-atom parameters constrained
Δρ_max_ = 0.33 e Å^−3^
Δρ_min_ = −0.46 e Å^−3^

### 

Data collection: *CAD-4 Software*; cell refinement: *CELDIM* (Enraf–Nonius, 1989 ▶); data reduction: *HELENA*/*PLATON* (Spek, 2009 ▶); program(s) used to solve structure: *SHELXS97* (Sheldrick, 2008 ▶); program(s) used to refine structure: *SHELXL97* (Sheldrick, 2008 ▶); molecular graphics: *SHELXTL* (Sheldrick, 2008 ▶); software used to prepare material for publication: *SHELXTL*.

## Supplementary Material

Crystal structure: contains datablock(s) global, I. DOI: 10.1107/S1600536811051828/qk2020sup1.cif

Structure factors: contains datablock(s) I. DOI: 10.1107/S1600536811051828/qk2020Isup2.hkl

Additional supplementary materials:  crystallographic information; 3D view; checkCIF report

## Figures and Tables

**Fig. 1.**
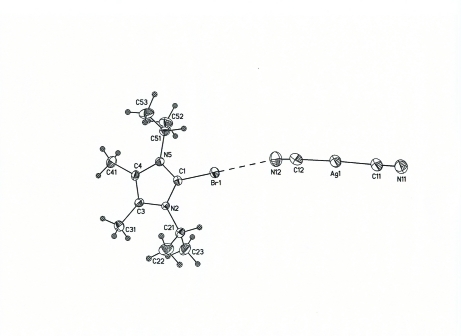
The molecular structure the cation-anion pair of the title compound showing 20% probability displacement ellipsoids for non-H atoms. The symmetry transformation for the depicted dicyanoargentate anion is x-1, y, z.

## supplementary crystallographic information

### Comment

N-heterocyclic carbenes can form stable coordination compounds with main group elements by using their strongly basic character. Kuhn *et al.* (2004) showed that weak interionic halogen contacts between 2-haloimidazolium cations and halogen containing counter anions do exist in the solid state. Results of related investigations were reported by Kuhn *et al.* (2009), Mallah *et al.* (2009), and Potocenak & Chomic (2006). The reaction of 2-bromo-1,3-diisopropyl-4,5-dimethylimidazolium bromide with silver cyanide gives the title compound as stable crystalline solid in a good yield. The structure contains an approximately C_2v_ imidazolium cation (Fig. 1), similar to the crystal structure of the 2-chloroimidazolium analogue (Mallah *et al.,* 2011), where the C—Cl bond (1.677 (5) Å) is shorter than the C—Br bond of the title compound (1.848 (6) Å). The title structure contains a cation-anion pair (Fig. 1) with a short N(12)(*x*-1,*y*,*z*)···Br(1) interaction of 2.96 (2) Å, by ca. 0.4 Å shorther than the sum of the van der Waals radii. Mascal *et al.* (1996) found for a s-triazine–dibromine cocrystal a N···Br contact distance as short as 2.515 Å.

### Experimental

The title compound was prepared by addition of silver cyanide (1.3 g, 9.7 mmol) to a solution of 2-bromo-1,3-diisopropyl-4,5-dimethylimidazoliumbromide, see: Kuhn *et al.* (2004), (1.1 g, 3.2 mmol) in 30 ml of acetonitrile. The mixture was stirred for 48 hr at room temperature, then the solvent was removed in vacuo and 20 ml of dichloromethane was added. The resulting solution was filtered off and solvent was removed in vacuo. Yield after recrystallisation from dichloromethane/diethyl ether 0.98 g (73 %), as colorless crystals.

### Refinement

There are no Friedel pairs because only the minimal data set was measured with the CAD4 instrument (±h, +k, +l). Hydrogen atoms were included at calculated positions with C—H = 0.95–1.00 Å and 1.5*U*_eq_(aliphatic C), using a riding-model approximation.

### Crystal data

**Table:**

(C_11_H_20_BrN_2_)[Ag(CN)_2_)]	*F*(000) = 832
*M**_r_* = 420.11	*D*_x_ = 1.635 Mg m^−^^3^
Orthorhombic, *P*2_1_2_1_2_1_	Mo *K*α radiation, λ = 0.71073 Å
Hall symbol: P 2ac 2ab	Cell parameters from 25 reflections
*a* = 6.6986 (15) Å	θ = 6.7–13.0°
*b* = 10.6222 (14) Å	µ = 3.52 mm^−^^1^
*c* = 23.989 (4) Å	*T* = 291 K
*V* = 1706.9 (5) Å^3^	Block, colourless
*Z* = 4	0.40 × 0.20 × 0.20 mm

### Data collection

**Table:**

Enraf–Nonius CAD-4 diffractometer	1781 reflections with *I* > 2σ(*I*)
Radiation source: fine-focus sealed tube	*R*_int_ = 0.052
graphite	θ_max_ = 26.4°, θ_min_ = 3.2°
ω scans	*h* = −8→8
Absorption correction: part of the refinement model (Δ*F*) (*DIFABS*; Walker & Stuart, 1983)	*k* = −1→13
*T*_min_ = 0.40, *T*_max_ = 1.00	*l* = −1→29
4327 measured reflections	3 standard reflections every 300 reflections
3475 independent reflections	intensity decay: 1.5%

### Refinement

**Table:**

Refinement on *F*^2^	Secondary atom site location: difference Fourier map
Least-squares matrix: full	Hydrogen site location: inferred from neighbouring sites
*R*[*F*^2^ > 2σ(*F*^2^)] = 0.052	H-atom parameters constrained
*wR*(*F*^2^) = 0.082	*w* = 1/[σ^2^(*F*_o_^2^) + (0.0199*P*)^2^] where *P* = (*F*_o_^2^ + 2*F*_c_^2^)/3
*S* = 0.98	(Δ/σ)_max_ = 0.001
3475 reflections	Δρ_max_ = 0.33 e Å^−^^3^
180 parameters	Δρ_min_ = −0.46 e Å^−^^3^
0 restraints	Extinction correction: *SHELXL97* (Sheldrick, 2008), Fc^*^=kFc[1+0.001xFc^2^λ^3^/sin(2θ)]^-1/4^
Primary atom site location: structure-invariant direct methods	Extinction coefficient: 0.00128 (16)

### Special details

**Table:**

Geometry. All esds (except the esd in the dihedral angle between two l.s. planes) are estimated using the full covariance matrix. The cell esds are taken into account individually in the estimation of esds in distances, angles and torsion angles; correlations between esds in cell parameters are only used when they are defined by crystal symmetry. An approximate (isotropic) treatment of cell esds is used for estimating esds involving l.s. planes.
Refinement. Refinement of F^2^ against ALL reflections. The weighted R-factor wR and goodness of fit S are based on F^2^, conventional R-factors R are based on F, with F set to zero for negative F^2^. The threshold expression of F^2^ > 2sigma(F^2^) is used only for calculating R-factors(gt) etc. and is not relevant to the choice of reflections for refinement. R-factors based on F^2^ are statistically about twice as large as those based on F, and R- factors based on ALL data will be even larger.

### Fractional atomic coordinates and isotropic or equivalent isotropic displacement parameters (Å^2^)

**Table:**

	*x*	*y*	*z*	*U*_iso_*/*U*_eq_	
Ag1	0.71941 (9)	0.98742 (7)	0.12744 (3)	0.0718 (2)	
C1	0.6144 (9)	0.5300 (5)	0.1252 (3)	0.0411 (16)	
N2	0.7155 (9)	0.5116 (6)	0.0780 (2)	0.0417 (16)	
C3	0.8807 (12)	0.4401 (6)	0.0898 (3)	0.045 (2)	
C4	0.8812 (11)	0.4187 (7)	0.1450 (3)	0.048 (2)	
N5	0.7109 (9)	0.4757 (6)	0.1669 (2)	0.0470 (16)	
C11	0.5572 (13)	1.1454 (10)	0.1123 (4)	0.072 (3)	
C12	0.8919 (14)	0.8303 (9)	0.1400 (4)	0.073 (3)	
C21	0.6467 (13)	0.5572 (8)	0.0225 (3)	0.059 (2)	
H21	0.5233	0.6041	0.0296	0.071*	
C22	0.7867 (15)	0.6484 (9)	−0.0028 (3)	0.092 (3)	
H22A	0.8289	0.7079	0.0249	0.138*	
H22B	0.7212	0.6921	−0.0327	0.138*	
H22C	0.9010	0.6044	−0.0171	0.138*	
C23	0.5874 (13)	0.4462 (9)	−0.0143 (3)	0.087 (3)	
H23A	0.7051	0.4020	−0.0260	0.131*	
H23B	0.5170	0.4766	−0.0465	0.131*	
H23C	0.5027	0.3902	0.0063	0.131*	
C31	1.0326 (11)	0.3991 (9)	0.0475 (3)	0.076 (3)	
H31A	1.1349	0.3508	0.0656	0.115*	
H31B	1.0910	0.4719	0.0303	0.115*	
H31C	0.9689	0.3484	0.0196	0.115*	
C41	1.0252 (11)	0.3428 (9)	0.1781 (3)	0.085 (3)	
H41A	1.1237	0.3072	0.1537	0.128*	
H41B	0.9553	0.2763	0.1969	0.128*	
H41C	1.0896	0.3957	0.2051	0.128*	
C51	0.6523 (12)	0.4807 (8)	0.2263 (3)	0.056 (2)	
H51	0.5282	0.5296	0.2266	0.067*	
C52	0.5934 (14)	0.3535 (9)	0.2481 (3)	0.089 (3)	
H52A	0.5176	0.3096	0.2202	0.133*	
H52B	0.5138	0.3635	0.2811	0.133*	
H52C	0.7112	0.3060	0.2569	0.133*	
C53	0.7912 (14)	0.5544 (8)	0.2608 (3)	0.086 (3)	
H53A	0.9073	0.5047	0.2688	0.129*	
H53B	0.7267	0.5776	0.2950	0.129*	
H53C	0.8301	0.6290	0.2410	0.129*	
N11	0.4683 (10)	1.2343 (8)	0.1047 (3)	0.076 (2)	
N12	0.9891 (11)	0.7474 (8)	0.1450 (4)	0.090 (3)	
Br1	0.37724 (11)	0.61731 (7)	0.13261 (3)	0.0596 (3)	

### Atomic displacement parameters (Å^2^)

**Table:**

	*U*^11^	*U*^22^	*U*^33^	*U*^12^	*U*^13^	*U*^23^
Ag1	0.0664 (4)	0.0772 (5)	0.0718 (4)	0.0139 (4)	0.0084 (4)	0.0021 (5)
C1	0.040 (4)	0.032 (4)	0.051 (4)	−0.001 (3)	0.002 (5)	−0.007 (5)
N2	0.040 (3)	0.053 (4)	0.033 (3)	0.010 (4)	−0.003 (3)	−0.001 (4)
C3	0.050 (5)	0.043 (5)	0.042 (5)	0.008 (4)	−0.005 (4)	−0.004 (4)
C4	0.037 (4)	0.052 (5)	0.054 (5)	0.003 (4)	−0.007 (4)	0.010 (4)
N5	0.046 (3)	0.053 (4)	0.042 (3)	0.003 (4)	−0.001 (3)	0.009 (4)
C11	0.070 (7)	0.077 (8)	0.069 (7)	0.017 (5)	0.009 (5)	−0.001 (6)
C12	0.077 (6)	0.069 (7)	0.072 (7)	−0.005 (5)	0.015 (6)	−0.009 (6)
C21	0.057 (5)	0.070 (6)	0.050 (5)	0.016 (5)	−0.005 (4)	0.010 (5)
C22	0.106 (8)	0.100 (9)	0.070 (6)	−0.029 (7)	−0.004 (6)	0.030 (6)
C23	0.081 (6)	0.108 (9)	0.073 (6)	0.010 (6)	−0.026 (6)	−0.013 (6)
C31	0.061 (5)	0.104 (8)	0.063 (5)	0.024 (6)	0.003 (4)	−0.011 (6)
C41	0.072 (6)	0.112 (9)	0.072 (6)	0.031 (6)	−0.012 (5)	0.023 (6)
C51	0.063 (6)	0.062 (6)	0.042 (4)	0.007 (6)	0.003 (4)	0.006 (5)
C52	0.104 (8)	0.100 (9)	0.061 (6)	−0.016 (7)	0.022 (6)	0.010 (6)
C53	0.112 (8)	0.094 (8)	0.052 (5)	−0.028 (7)	−0.008 (5)	−0.013 (5)
N11	0.069 (5)	0.079 (6)	0.079 (5)	0.009 (5)	0.001 (4)	−0.006 (5)
N12	0.082 (6)	0.071 (6)	0.117 (8)	0.005 (5)	0.002 (5)	−0.001 (6)
Br1	0.0552 (5)	0.0610 (5)	0.0624 (5)	0.0161 (4)	0.0033 (5)	−0.0025 (6)

### Geometric parameters (Å, °)

**Table:**

Ag1—C11	2.032 (10)	C22—H22C	0.9600
Ag1—C12	2.053 (10)	C23—H23A	0.9600
C1—N5	1.325 (7)	C23—H23B	0.9600
C1—N2	1.332 (7)	C23—H23C	0.9600
C1—Br1	1.848 (6)	C31—H31A	0.9600
N2—C3	1.371 (8)	C31—H31B	0.9600
N2—C21	1.490 (8)	C31—H31C	0.9600
C3—C4	1.344 (8)	C41—H41A	0.9600
C3—C31	1.501 (10)	C41—H41B	0.9600
C4—N5	1.394 (8)	C41—H41C	0.9600
C4—C41	1.486 (9)	C51—C53	1.471 (11)
N5—C51	1.478 (8)	C51—C52	1.502 (11)
C11—N11	1.131 (10)	C51—H51	0.9800
C12—N12	1.102 (10)	C52—H52A	0.9600
C21—C22	1.479 (10)	C52—H52B	0.9600
C21—C23	1.526 (10)	C52—H52C	0.9600
C21—H21	0.9800	C53—H53A	0.9600
C22—H22A	0.9600	C53—H53B	0.9600
C22—H22B	0.9600	C53—H53C	0.9600
			
C11—Ag1—C12	177.5 (4)	C21—C23—H23C	109.5
N5—C1—N2	109.2 (5)	H23A—C23—H23C	109.5
N5—C1—Br1	124.4 (6)	H23B—C23—H23C	109.5
N2—C1—Br1	126.4 (6)	C3—C31—H31A	109.5
C1—N2—C3	108.5 (5)	C3—C31—H31B	109.5
C1—N2—C21	123.6 (6)	H31A—C31—H31B	109.5
C3—N2—C21	127.9 (6)	C3—C31—H31C	109.5
C4—C3—N2	107.4 (7)	H31A—C31—H31C	109.5
C4—C3—C31	127.9 (8)	H31B—C31—H31C	109.5
N2—C3—C31	124.6 (6)	C4—C41—H41A	109.5
C3—C4—N5	107.2 (7)	C4—C41—H41B	109.5
C3—C4—C41	128.3 (8)	H41A—C41—H41B	109.5
N5—C4—C41	124.4 (6)	C4—C41—H41C	109.5
C1—N5—C4	107.7 (5)	H41A—C41—H41C	109.5
C1—N5—C51	125.7 (6)	H41B—C41—H41C	109.5
C4—N5—C51	126.6 (6)	C53—C51—N5	113.2 (7)
N11—C11—Ag1	178.8 (9)	C53—C51—C52	116.7 (7)
N12—C12—Ag1	177.2 (11)	N5—C51—C52	111.9 (7)
C22—C21—N2	112.6 (7)	C53—C51—H51	104.5
C22—C21—C23	115.7 (7)	N5—C51—H51	104.5
N2—C21—C23	110.3 (6)	C52—C51—H51	104.5
C22—C21—H21	105.8	C51—C52—H52A	109.5
N2—C21—H21	105.8	C51—C52—H52B	109.5
C23—C21—H21	105.8	H52A—C52—H52B	109.5
C21—C22—H22A	109.5	C51—C52—H52C	109.5
C21—C22—H22B	109.5	H52A—C52—H52C	109.5
H22A—C22—H22B	109.5	H52B—C52—H52C	109.5
C21—C22—H22C	109.5	C51—C53—H53A	109.5
H22A—C22—H22C	109.5	C51—C53—H53B	109.5
H22B—C22—H22C	109.5	H53A—C53—H53B	109.5
C21—C23—H23A	109.5	C51—C53—H53C	109.5
C21—C23—H23B	109.5	H53A—C53—H53C	109.5
H23A—C23—H23B	109.5	H53B—C53—H53C	109.5
			
N5—C1—N2—C3	1.5 (7)	Br1—C1—N5—C51	−3.3 (10)
Br1—C1—N2—C3	−178.6 (5)	C3—C4—N5—C1	−0.5 (8)
N5—C1—N2—C21	178.3 (6)	C41—C4—N5—C1	−177.2 (7)
Br1—C1—N2—C21	−1.8 (9)	C3—C4—N5—C51	−177.7 (7)
C1—N2—C3—C4	−1.8 (8)	C41—C4—N5—C51	5.7 (12)
C21—N2—C3—C4	−178.5 (7)	C12—Ag1—C11—N11	−100 (52)
C1—N2—C3—C31	180.0 (7)	C11—Ag1—C12—N12	−5(30)
C21—N2—C3—C31	3.3 (12)	C1—N2—C21—C22	118.1 (8)
N2—C3—C4—N5	1.4 (8)	C3—N2—C21—C22	−65.7 (10)
C31—C3—C4—N5	179.5 (7)	C1—N2—C21—C23	−111.1 (8)
N2—C3—C4—C41	177.9 (7)	C3—N2—C21—C23	65.2 (10)
C31—C3—C4—C41	−4.0 (14)	C1—N5—C51—C53	−111.4 (8)
N2—C1—N5—C4	−0.6 (8)	C4—N5—C51—C53	65.2 (11)
Br1—C1—N5—C4	179.5 (5)	C1—N5—C51—C52	114.3 (9)
N2—C1—N5—C51	176.6 (7)	C4—N5—C51—C52	−69.0 (10)
